# 

**Published:** 1914-06

**Authors:** 


### CONTENTS.

ORIGINAL COMMUNICATIONS—

Injuries of the Kidney. By Frank K. Boland, M. D., Atlanta, Ga. ---- 97

Case-Histories in Pyorrhea Alveolaris. By Wm. Crenshaw, D. D. S., Atlanta, Ga. ---- 107

Fractures of Lower End Humerus Involving the Elbow. By W. E. Person, Atlanta, Ga. ---- 111

Certified versus Pasteurized Milk. By C. W. Gould, M. D. ---- 115

EDITORIAL ---- 122

SELECTIONS AND ABSTRACTS ---- 125

